# Assessing the Impact of Neighborhood Conditions on Neurodevelopmental Disorders during Childhood

**DOI:** 10.3390/ijerph18179041

**Published:** 2021-08-27

**Authors:** Anna Maria Santiago, Kristen A. Berg, Joffré Leroux

**Affiliations:** 1 School of Social Work, College of Social Science, Michigan State University, East Lansing, MI 48824, USA; 2 Center for Health Care Research and Policy, Case Western Reserve University, Cleveland, OH 44106, USA; kristen.berg@case.edu; 3 Department of Economics, Michigan State University, East Lansing, MI 48824, USA; lerouxjo@msu.edu

**Keywords:** neurodevelopmental disorders, neighborhood effects, natural experiments, IV probit estimation

## Abstract

Nearly three out of ten neurodevelopmental disabilities in the United States have been linked to environmental conditions, prompting emerging lines of research examining the role of the neighborhood on children’s developmental outcomes. Utilizing data from a natural experiment in Denver, this study quantifies the impact of exposure to varied neighborhood contexts on the diagnosis of neurodevelopmental disorders over the course of childhood. Our analysis is based upon retrospective child, caregiver, household and neighborhood data derived from the *Denver Child Study* for a sample of approximately 590 Latino and African American children and youth whose families were quasi-randomly assigned to subsidized housing operated by the Denver (CO) Housing Authority during part of their childhood. We employed binary response models with endogenous explanatory variables, estimated using instrumental variables (IV) probit and average marginal effects to identify predictors of a neurodevelopmental disorder diagnosis during childhood. We found that multiple dimensions of neighborhood context—especially neighborhood socioeconomic status, older housing stock, residential instability and prevalence of neurological hazards in the ambient air—strongly and robustly predicted the diagnosis of a neurodevelopmental disorder during childhood.

## 1. Introduction

Since the National Research Council of the United States reported in 2000 that more than 28% of neurodevelopmental disabilities have an environmental origin, why some children have inferior developmental outcomes has been the subject of heightened scholarly study and debate. Though heritable and household contextual factors are undoubtedly vital, some argue that both physical (“integral”) and social (“contextual”) dimensions of the environment—the oft-labeled “neighborhood effects” literature—generate impacts [1,2]. This vast interdisciplinary literature has long grappled with the thorny questions of how such neighborhood effects on health might arise and whether they are substantively large or clinically significant [3,4,5,6,7,8]. Nonetheless, discerning how and why certain neighborhood circumstances affect specific health outcomes remains a critical realm of current and future research [4,6,7,8,9,10,11].

Neighborhoods may affect children’s development through a variety of causal mechanisms operating either through social, institutional or biological processes; for extended discussions see Sampson, Morenoff and Gannon-Rowley [12] and Galster [11]. The potential neighborhood social mechanisms relevant for neurodevelopmental outcomes include: neighborhood deprivation [13,14]; social cohesion and control [15]; ethnic mix [16]; violence and social disorder [17]; institutional resources [18]; physical surroundings [19,20]; and exposure to environmental pollutants [6,9,21,22,23]. These mechanisms may affect health outcomes via biological responses and/or alterations in health and risk behaviors and usage of health facilities.

Several multivariate statistical studies based on individual observations, and adjusted for relevant individual and parental characteristics, have explored the association between aspects of neighborhood physical, social and demographic environment (typically operationalized as a census tract) and children’s various neurodevelopmental disorders and cognitive outcomes. Numerous studies have documented statistical associations between such outcomes and concentrations of neurotoxins in the environment. Key neurotoxins investigated have been various particulate matter components of air pollution [24,25,26], lead and heavy metal exposures [27,28,29,30,31,32,33,34] and pesticides [35,36,37,38,39,40,41,42].

Another substantial body of research has identified associations between children’s poorer cognitive outcomes and the low socioeconomic position of their neighborhoods [14,38,43,44,45,46,47,48,49,50,51]. For instance, studies have linked higher numbers of caregiver-reported physical neighborhood hazards [52] and lower levels of neighborhood social support [15] with greater incidence of attention deficit hyperactivity disorder. In a European sample, neighborhood-level socioeconomic deprivation was associated with higher prevalence of autism spectrum disorders with co-occurring intellectual disability [53]. Most recently, Vargas et al. [51] found neighborhood socioeconomic deprivation to predict poorer neurocognitive performance among late childhood and early adolescent youth, net of parent and household factors, implicating neighborhood-level socioeconomic circumstances as a key environmental vulnerability for children’s neurodevelopment.

The causal interpretation of the associations measured by these studies is, however, clouded by the geographic selection bias problem; for methodological reviews, see Sampson, Morenoff and Gannon-Rowley [12] and Galster [54]. The problem of inferring causation arises because parents or primary caregivers may possess unmeasured characteristics, statistically uncontrolled for by the researcher, that strongly influence both where their household resides and developmental outcomes for their children. It is therefore uncertain whether any observed associations between neighborhood characteristics and child outcomes are causal or merely spuriously related with both being produced by unobserved parental characteristics. In his critiques of the neighborhood effects on health literature, Oakes [10] and colleagues [8] concluded that the only sources of reliable causal inferences about neighborhood effects on health are studies based on random-assignment experiments or natural quasi-experiments that mimic the random assignment of households to neighborhoods. To our knowledge, there has been only one such experimental study conducted to date [55] (a systematic Boolean search, “(randomized control trial or RCT or randomised control trial or (randomized controlled trial) and (neighborhood effects),” was run in order to ensure inclusion of all relevant literature published through May, 2021. Databases searched included PsycInfo, CINAHL, MEDLINE, Psychology and Behavioral Sciences Collection, and SocINDEX. (Information from the first two sources applied to census tracts; Piton data applied to well-defined Denver neighborhood areas approximately two census tracts in size; caregiver responses referred to the “neighborhood” as they defined it.). However, this study did not directly investigate neurodevelopmental disorders.

In a random-assignment experiment, data are produced by an experimental design whereby households are randomly assigned to different neighborhoods. The only extant example of a random assignment experiment relevant to the health field is the well-known Moving To Opportunity (MTO) demonstration project [55]. The MTO experiment involved assigning low-income (primarily African American) residents of public housing in socioeconomically deprived neighborhoods to one of three groups: (a) control, wherein families received no rental housing vouchers but remained eligible for any other entitled assistance; (b) rental housing voucher, wherein families received vouchers to use for any relocation but no housing counseling; or (c) voucher plus relocation counseling, wherein families received both housing vouchers and relocation counseling plus a requirement to live in low-poverty neighborhoods for at least the first year of the program. Neurocognitive developmental disorders were not investigated directly; however, considering academic achievement as somewhat of a proxy, results showed no substantial differences in academic achievement measures between the experimental and control groups and only a minor difference for girls on an emotional–behavioral problems index [55]. The MTO findings are provocative, but they leave many unanswered questions about neighborhood impacts on children’s neurodevelopment because of several methodological and operational shortcomings [56,57].

In natural quasi-experiments, observations are produced by idiosyncratic housing policy interventions, such as subsidized housing desegregation or revitalization programs, or inclusionary zoning regulations, which create exogenous variation in neighborhood environments for assisted tenants [54]. Although such approaches have been employed to explore a variety of outcomes, to our knowledge it has never been used in the realm of child neurodevelopmental outcomes. As a result, the existing literature has not provided definitive evidence about the potential child developmental benefits (in this case, reduced incidence of neurodevelopmental disorders) from sustained residence in neighborhoods that are advantaged on multiple social and physical dimensions. Moreover, little is known about the effects that these approaches might have on outcomes for low-income Latino and African American children.

The current study represents the first such approach in this field. Our study hopes to contribute by leveraging a natural quasi-experiment associated with the Housing Authority of the City and County of Denver (DHA). In this paper we quantify the impact of exposure to varied neighborhood contexts on the diagnosis of neurodevelopmental disorders (defined here as intellectual disabilities, learning disabilities, developmental delays, autism, and attention deficit disorder (ADD) or attention deficit hyperactivity disorder (ADHD)) for low-income Latino and African American children. Specifically, we address the following research question:

For Latino and African American children who spent at least two years of their childhood living in subsidized housing, are there statistically and substantively significant differences in the diagnosis of neurodevelopmental disorders attributable to differences in their childhood neighborhood socioeconomic, demographic, safety and physical environments, all else equal?

Our work advances the literature on quantifying the neighborhood’s causal impact on low-income, minority children’s development in two ways. First, because caregivers of our sampled children were quasi-randomly assigned to neighborhoods, the challenge of parental geographic selection bias is addressed. Second, we evaluate an unprecedented variety of measures of neighborhood environment (both social and physical), measured at different spatial scales, which vary substantially across the sample.

## 2. Materials and Methods

### 2.1. The Natural Experiment Involving Public Housing in Denver

In addition to its 3000 units in large-scale, conventional public housing developments, the DHA has operated since 1969 a dispersed housing program providing approximately 1500 low-income families with opportunities to live in scattered-site, single-family and small-scale, multi-family units. These dispersed units are located in a wide range of neighborhoods throughout the congruent City and County of Denver, whereas the conventional developments are typically more concentrated. From 1987 to 2005, as applicants came to the top of the public housing waitlist, they were offered a vacant DHA unit with the number of bedrooms appropriate for their family size and gender of children. If offered units were refused, applicants were dropped to the bottom of the waitlist. As detailed in Santiago and colleagues [58], we have conducted a variety of statistical investigations that enabled us to reach the conclusion that initial assignment of families to a DHA dwelling unit and neighborhood mimicked random assignment of families to neighborhood, conditioned on ethnicity (which we control in our analyses). 

Another important feature of our natural experiment is the comparatively long exposures children in DHA families had to their assigned neighborhoods. Our study sample had a 6-year mean (median = 5 years) duration in DHA subsidized housing, approximately twice as long as reported for the MTO experimental group (mean = 2.7 years; median = 3.3 years). Previous work by Wodtke, Harding and Elwert [59] and Crowder and South [60] underscores the importance of accounting for the duration of exposure to particular neighborhood contexts to avoid underestimating the true effects that neighborhoods have on child outcomes.

### 2.2. Analytical Approach Utilizing the Natural Experiment

Our analytical approach for causal identification exploits the quasi-experimental assignment that produces exogenous variation in neighborhood context. Specifically, the “treatment” that the DHA offers to its families is a complex bundle of neighborhood attributes (the components of which we describe below). We test the consequences of these various treatments by employing binary response models with endogenous explanatory variables, estimated using instrumental variables (IV) probit to calculate the average marginal effects of neighborhood attributes on the probability of ever being diagnosed with a neurodevelopmental disorder during childhood. We only consider children’s initial diagnoses occurring after their families have been quasi-randomly assigned to a DHA public housing unit, thereby preserving the value of the natural experiment for drawing causal inferences. First-offer neighborhood characteristics are used as instruments for estimating neighborhood characteristics at time of initial diagnosis.

Our analytic approach shares much in common with the MTO study. Low-income families are (quasi-) randomly assigned to different neighborhood contexts and are then permitted to move as they wish. In the case of the MTO experimental group, such moves occurred after a minimum one-year stay in a low-poverty area. Several years later, researchers retrospectively analyze whether children exposed to these different contexts behaved differently, controlling for characteristics of their families. As in MTO, we do not view potential residential moves from the originally assigned DHA dwelling as a source of geographic selection bias, but rather as a potential impact of the original treatment.

### 2.3. Denver Child Study

Analysis samples are drawn from the *Denver Child Study*, a retrospective study of Latino and African American, current or former Denver (CO) Housing Authority (DHA) residents whose families were randomly assigned to subsidized housing units and neighborhoods during the period between 1987 and 2005 (for more details see Supplementary Material and Santiago et al. [58]). Children in the sample were restricted to those under the age of 18 who resided with their families at the time of random assignment and lived in DHA housing for two or more years during childhood. Further, we restricted the sample to those children who were diagnosed with one or more neurodevelopmental disorders since initial random assignment to neighborhoods but before the age of 18. This produced a final analysis sample of 588 children.

In the *Denver Child Study*, caregivers were asked about a variety of adverse health issues with the question “Has a doctor or medical professional ever diagnosed your child with the following…If so, in what year was this first diagnosed?” The outcome variables of interest here are: (1) whether the child, by the time of our survey (or by 18 if this age or older at time of survey), had been clinically diagnosed with one or more neurodevelopmental disorders: mental retardation or intellectual disabilities, learning disabilities, developmental delays, autism, ADD or ADHD; and (2) the child’s age at time of diagnosis or time of survey or age 18 if not diagnosed. Approximately 7% of our sampled children were diagnosed with neurodevelopmental disorders since moving into DHA, with a mean age of diagnosis at 6.5 years (range 2–14 years); see Table 1.

We recognize that there is ambiguity in our neurodevelopmental disorder indicator, as is the case in most community health investigations. A diagnosis outcome results from the joint probabilities that a child exhibits adverse health symptoms, and that the caregiver will seek medical advice given the symptoms. The latter probability, in turn, is a function of the caregiver’s physical and mental health, personal efficacy, economic resources and insurance, but we control for these in our models. It also depends on the institutional structure that the caregiver can access easily, such as proximity to medical facilities. Neighborhood context potentially can affect some or all of the aforementioned components leading to a diagnosis. Unfortunately, we are unable to discern these mechanisms, though we do attempt to measure neighborhood institutional resources and medical facilities. Thus, we emphasize that our estimated statistical associations represent a “net impact” of the given neighborhood indicator on the odds of a diagnosis, not necessarily on the odds that the child has a neurodevelopmental disorder. The implication is that our findings here need to be interpreted with care: a statistical association should not be viewed as unambiguously good or bad normatively, regardless of its sign. If certain neighborhood attributes are, for example, associated with higher odds of a neurodevelopmental disorder diagnosis, this may be “bad” because it indicates that these places are less healthy for children. Yet, this finding may be “good” because it indicates that children who have a neurodevelopmental disorder are more likely to be diagnosed and treated.

Our *Denver Child Study* survey collected information on a wide variety of child, caregiver, and household characteristics that we employed as controls; these are summarized in Table 1 and described in more detail in Supplementary Material and Santiago and colleagues [58]. All time-varying characteristics were measured at the time of first DHA offer. Approximately 58% of the study sample children and youth were Latino and 42% were African American. A slight majority (53%) were male. At time of the first DHA offer, caregivers were, on average, 30.5 years old, 16% were immigrants, and 44% held a high school diploma or higher. One out of 20 caregivers indicated they had one or more disabilities. The average household at the time of the first DHA offer had 1.2 siblings and is used as an indicator of crowding, although federal guidelines require assignment to units with sufficient bedrooms to meet household needs. Mean household income was $10,164 but ranged from $0 to $39,520. The average household had experienced at least one of the following household stressors: caregiver unemployment, major illness or injury, insufficient food, utility shutoffs, or eviction. Additionally, the household had experienced, on average, 2.4 moves prior to receiving their first DHA offer.

### 2.4. Estimation of Neighborhood Indicators

Once residential history information obtained on the survey was verified for accuracy, we geo-coded each address using the U.S. Bureau of the Census’s *American FactFinder* website utility. We successfully linked 92% of the residential locations listed by respondents with a rich set of neighborhood indicators obtained from four sources: (1) the U.S. Census via the *Neighborhood Change Data Base* (a Geolytics proprietary product); (2) the U.S. Environmental Protection Agency; (3) the Piton Foundation Neighborhood Facts Database; and (4) caregiver responses from the *Denver Child Study*. Details of the data sources and indicator construction are described in Supplementary Material; see also Santiago and colleagues [58]. The neighborhood characteristics of interest in this study include indicators of neighborhood disadvantage and affluence, ethnic composition, residential instability, exposure to violence, housing stock, and exposure to ambient neurological air toxicants.

To get a sense of the sorts of places where our sampled children resided when they had their first diagnosis of a neurodevelopmental disorder, we present the mean characteristics of their neighborhoods in Table 2. We underscore, however, that it is the substantial variation around the mean that gives our study unusual power. Relative to the typical child residing in the City and County of Denver as a whole, children in our full sample resided in neighborhoods with lower prestige and whose levels of social vulnerability were 37% higher. On average, study children lived in neighborhoods with 10% higher property crime rates. Their neighborhoods experienced considerable residential turnover (26%), and, on average, one-quarter of the housing units were built before 1940. Neighborhood ambient neurological hazards were derived from the U.S. Environmental Protection Agency National Air Toxics Assessments that provide emissions data, ambient concentrations and health effects associated with outdoor air quality. The neurological hazards index traces exposure to 23 toxins ranging from lead compounds, mercury, styrene, and solvents that have been linked to neurological and developmental disorders The index is measured in parts per million but have been rescaled by 100 for our analyses [61]. The average neighborhood neurological hazards index at time of diagnosis was approximately 4% higher than for Denver as a whole. Thus, it is not surprising that their neighborhoods exhibited more environmental hazards.

### 2.5. Analytical Models

We merged the aforementioned information regarding sampled households, caregivers, children, and their corresponding neighborhood environments to create a pseudo-longitudinal database wherein the *child-year* becomes the unit of analysis. We estimate the probability of individual *i* living in neighborhood *j* of ever being diagnosed with a neurodevelopmental disorder using instrumental variables (IV) probit estimation and the *ivprobit* and *margins commands* in Stata 14.0 (StataCorpLP, College Station, TX, USA). IV probit allows the consistent estimation of a binary response model in the presence of endogeneity in some of the regressors. We name $y_{1i}$ the observed binary outcome variable and $y_{1i}^{*}$ the latent variable determining the value of $y_{1i}$.

(1) $$y_{1i}^{*}=y_{2ij}\beta +x_{1i}\gamma +u_{i}$$

(2) $$y_{2ijk}=x_{1i}\delta_{1k}+x_{2ij}\delta_{2k}+v_{ik}$$

(3) $$y_{1i}=\{\begin{array}{c} 0\;if\;y_{1i}^{*}<0\\ 1\;if\;y_{1i}^{*}\geq 0 \end{array}$$

$y_{2ij}$ is a vector of neighborhood characteristics at time of initial diagnosis, believed to possibly be endogenous, and $x_{1i}$ a vector of time-invariant demographic characteristics, caregiver and household characteristics at the time of initial diagnosis, and a set of dummy variables denoting the calendar year when the offer was made.

We use this model in order to account for the endogeneity that could be caused by households choosing to live in a neighborhood other than the one to which they were initially randomly assigned. For each potentially endogenous neighborhood characteristic $y_{2ijk}$ in $y_{2ij}$, we use the same set of explanatory variables $x_{1i}$ along with a vector of instruments $x_{2ij}$ that includes the same set of neighborhood characteristics measured at the time of first offer for the randomly assigned neighborhood. Parameters are jointly estimated using maximum likelihood estimation and standard errors are robust to clustering within households. We assume $\left(u_{i},v_{ik}\right)$ are normally distributed and independent of all x.

Average marginal effects (AMEs) were calculated for each explanatory variable. For each variable and each individual in the sample, the marginal effect was calculated by finding the change in predicted probability if that variable increased by one, holding other individual characteristics constant, and marginal effects for all individuals were averaged across the sample. Delta method standard errors were adjusted for clustering within households. Continuous independent variables were standardized for ease of interpretation such that MEs correspond to a one standard deviation increase in the measure above the mean value.

Although this is not a traditional two-stage least-squares model, the same requirements of relevance and exogeneity on instruments apply in order for the estimates to have a causal explanation. As we have argued previously (see Galster et al. [62]), we believe that these instruments are valid because there is no plausible reason why the offer of a neighborhood or the year of such offer should: (a) influence children’s neurodevelopmental outcomes other than through their relationship with the actual neighborhood context experienced; or (b) be related to unobservable caregiver characteristics related to both their neighborhood preferences and their children’s neurodevelopmental outcomes.

In the first stage regressions obtained from estimating the model using a two-step procedure on the full sample, F-statistics ranged from 6.59 to 18.59, with five falling below the suggested rule of thumb threshold of 10 proposed by Staiger and Stock [63]. However, they note that F-statistics below their proposed threshold does not imply that the results are not statistically significant or theoretically important.

Given our binary IV probit models, classical residual analysis was not well suited for residual plots [64]. Instead, we report an alternative goodness of fit measure based on predictive accuracy. We calculated the percent correctly predicted as suggested by Wooldridge [65] by comparing each observation’s predicted probability of being diagnosed with a neurodevelopmental disorder during childhood to its actual outcome. We label an observation as “correctly predicted” if its predicted probability of diagnosis is greater than the share of individuals in the sample who are correctly diagnosed. In the full sample, the share of individuals diagnosed with a neurodevelopmental disorder is 0.066. Nearly three-quarters of the observations (73.3%) were correctly predicted, with more false positives than negatives. Approximately 26% of the observations have a predicted probability of greater than 0.066 but were not diagnosed while 1% have a predicted probability of less than 0.066 but were diagnosed.

## 3. Results

The results for average marginal effects (AMEs) from our IV probit estimation predicting initial diagnosis of a neurodevelopmental disorder during childhood are presented in Table 3. Since we are interested in neighborhood predictors of that diagnosis, our discussion will focus on those elements of the physical and social neighborhood that increase the likelihood of a diagnosis. Normalized versions of all continuous variables are employed to aid cross-predictor comparisons. Overall model performance is acceptable according to the Wald chi-square criteria.

### Neighborhood-Level Predictors of Neurodevelopmental Disorder Diagnosis during Childhood

Of more central interest to our study, a number of contemporaneous neighborhood indicators related to social vulnerability and prestige, residential instability, age of housing stock and pollution were robust, statistically significant predictors of a neurodevelopmental disorder diagnosis during childhood. In overview, for a one standard deviation-higher neighborhood:

1. Social vulnerability was associated with a 40.6 percentage point increase in the probability of being diagnosed;
2. Occupational prestige scale was associated with an 18.3 percentage point increase in the probability of being diagnosed;
3. Resident instability was associated with a 12 percentage-point decrease in the probability of being diagnosed;
4. The proportion of housing stock built before 1940 was associated with an 18.5 percentage-point increase in the probability of being diagnosed;
5. The neurological hazards index was associated with a 42.7 percentage-point decrease in the probability of being diagnosed.

## 4. Discussion

In overview, the results reported above clearly show that many aspects of neighborhood physical and social context are statistically and substantively important predictors of a neurodevelopmental disorder diagnosis during childhood. Since they are produced in a natural experiment mimicking random assignment, we will discuss them as if they were indicative of causal effects. Below, we organize the discussion around thematic categories of neighborhood social context.

### 4.1. Neighborhood Social Status

Neighborhood social vulnerability and occupational prestige proved strongly predictive of increased diagnosis of a neurodevelopmental disorder during childhood, consistent with the aforementioned empirical literature on neighborhood socioeconomic status and child development. Consistent with prior studies [13,14], our findings suggest that children in neighborhoods with higher levels of disadvantage are more likely to be diagnosed with neurodevelopmental disorders. While previous studies have underscored the higher prevalence of neurodevelopmental disorders among children residing in disadvantaged neighborhoods, the association with residence in more prestigious neighborhoods has not. In contrast to prior studies, we also found that children in more affluent neighborhoods were more likely to be diagnosed with a neurodevelopmental disorder which may reflect greater awareness of such disorders and access to institutional resources that could provide such diagnoses. This positive association with neighborhood occupational prestige has intuitive appeal from the perspective of local information networks, norms and role models related to pro-child development behaviors of caregivers and their children. We, thus, are persuaded that the occupation prestige result provides evidence of an unambiguously pro-developmental neighborhood effect.

### 4.2. Neighborhood Physical Environment

We also found evidence that older housing in the neighborhood may provide a less sanguine environment in which to raise children. We cannot know from our study whether this association may be due to the environmental conditions inside these older houses and yards (such as the presence of lead paint) in which neighbor children may conduct much of their routine activities or whether associated urban density and design features in older Denver neighborhoods may be responsible. Nonetheless, the positive association between higher fractions of older housing and a neurodevelopmental disorder diagnosis supports conventional medical wisdom regarding the deleterious consequences of exposure to toxicants for child development (see review in Emerson [6]).

Although we found a strong, significant negative association between the presence of higher levels of neurological hazards and diagnosis of a neurodevelopmental disorder, we believe this may be an instance where caregivers and others residing in these neighborhoods might see their children’s behaviors as normative or, unlike asthma, are less likely to associate ambient air quality with the formation of such disorders. Additionally, while young children are more likely to be tested for lead poisoning, they may not be as likely to be tested for effects of other air toxicants unless they have asthma. Diagnoses of neurodevelopmental disorders also tend to be made once children start attending elementary school where intellectual, developmental, or behavioral difficulties are more likely to be noticed and thus tested.

In an earlier work [58], we also examined the potential role that neighborhood parks, playgrounds and recreational centers as well as other resources might play in a neurodevelopmental disorder diagnosis during childhood. Although we acknowledge their importance in creating healthy environmental contexts for children, they were never found to be statistically significant predictors of a neurodevelopmental disorder diagnosis.

### 4.3. Neighborhood Instability

Our findings found an intriguing relationship between neighborhood instability and the probability of a neurodevelopmental disorder diagnosis during childhood. Children residing in neighborhoods experiencing a considerable churn of residents were less likely to be diagnosed with a neurodevelopmental disorder. While the literature has discussed links between the residential stability of the child and these disorders, there has been little discussion about the role that resident churn might play in delaying such diagnoses. Perhaps this reflects a decrease in connections to neighborhood information networks, weakened norms and social ties to neighbors—connections which might facilitate knowledge about and help-seeking for such disorders, as well as provide social support to affected children and their families [15].

## 5. Conclusions, Limitations and Implications

Community health providers, practitioners and policymakers have a professional obligation to encourage the development and maintenance of economically and ethnically diverse neighborhoods and expand opportunities for disadvantaged citizens. Our research provides further impetus for progress toward these goals because it demonstrates that the most fundamental, indispensable cognitive benefits would redound to low-income Latino and African American children. We make this strong assertion because we have an unusual opportunity to measure child context-developmental associations that are plausibly produced by causal processes. An innovative public housing program instituted by the Denver Housing Authority provides a unique opportunity to explore this issue because the DHA mimics a random assignment to a wide range of neighborhoods for families with children who apply for DHA housing as evidenced by the variations from the average of our indicators, thereby enabling us to examine potential effects in disadvantaged and affluent neighborhoods. Moreover, families typically reside in DHA housing for nontrivial periods, thus producing sustained exposure to context.

We employed IV probit estimation and calculated average marginal effects to determine the predictors of a low-income minority child’s initial diagnosis of a neurodevelopmental disorder. Several aspects of neighborhood social (social status, residential churn) and physical (older housing stock, neurological hazards index) contexts proved statistically and substantively important predictors of an initial diagnosis. We caution, however, that it is sometimes unclear whether these associations were manifested by causal links though the probability of a child exhibiting adverse neurodevelopmental symptoms, or through the probability of having presented symptoms that were medically diagnosed.

Although we contend that our approach offers important advances in providing convincing evidence of causal connections between neighborhood social context and child neurodevelopment, we acknowledge that our study has weaknesses. The first shortcoming is that our measure of child health is retrospectively reported by caregivers. We recognize that this can yield random errors associated with the timing of initial diagnosis, though we have no reason to suspect rampant inaccuracies in reporting whether the diagnosis occurred. To the extent that these random recall errors affected our data, they would push the neighborhood effect findings toward null. Second, although we have an unusually comprehensive set of neighborhood indicators, we recognize their limitations in describing the physical environs in and around the home in particular. Third, we have not attempted to probe in this paper potential pathways through which neighborhood environment may affect children’s development, especially as they might unfold through exposure to violence and a variety of other behaviors [21]. Fourth, we have not attempted to measure cumulative exposure to neighborhood social context. These additional analyses are constrained by the limitations of our sample size.

Despite these caveats, our results clearly suggest that healthcare providers, educators, community practitioners, and policymakers should be cognizant of both the social and physical aspects of neighborhoods as important developmental contexts affecting children’s health. Given our study design, we are confident in claiming that neurodevelopmental disorders are influenced by more than personal, family, dwelling or physical environmental characteristics of low-income, minority children. The daunting policy challenge is delineating how to best encourage the development and preservation of safe, diverse neighborhood environments that can be more developmentally nurturing for all children [66,67].

## Figures and Tables

**Table 1:** Child, Parent and Household Characteristics.

Characteristic	Full Sample			
	Mean or Percent	Standard Deviation	Minimum	Maximum
Ever diagnosed with a neurodevelopmental disorder (%)	6.6	24.9	0	1
Age of diagnosis for those diagnosed since DHA move-in	6.46	2.65	2	14
**Individual characteristics**				
Latina female (%)	27.4	44.6	0	1
Latino male (%)	30.8	46.2	0	1
African American female (%)	19.2	39.4	0	1
African American male (%)	22.6	41.9	0	1
**Parent/caregiver (PC) characteristics at time of first offer**				
PC age	30.5	8.8	16.2	67.9
PC has HS education or more (%)	44.2	49.7	0	1
PC has disability	5.3	0.2	0	1
PC is immigrant	15.6	36.4	0	1
**Household characteristics at time of first offer**				
Number of siblings in household	1.21	1.27	0	5
Household income ($)	10,164	10,338	0	39,520
Household stressor scale (0–5)	0.88	1.05	0	4
Mean number of moves between birth and first offer	2.08	2.44	0	12
*N*	588			

NOTE: *N* = 39 is the number of diagnosed cases for the full sample. Diagnoses were only counted if they occurred after the initial move-in to DHA housing.

**Table 2:** Neighborhood Characteristics at Time of First Offer, Time of Diagnosis and for the City and County of Denver.

	Full Sample			
	Mean or Percent	Standard Deviation	Minimum	Maximum
**Neighborhood characteristics at time of first offer**				
African American residents (%)	15.6	0.2	0.5	85.4
Foreign-born residents (%)	19.9	10.2	4.0	47.8
Social vulnerability index (0–400)	168.5	64.4	44.2	286.7
Occupational prestige score (29–62)	36.0	3.1	31.5	47.6
Property crime rate	90.5	51.8	4.9	357.3
Residents who moved in the preceding 12 months (%)	29.2	7.6	12.8	46.6
Housing built before 1940 (%)	28.7	17.9	0.1	70.9
Neurological hazards index (rescaled by 100)	8.6	1.9	5.3	17.9
**Neighborhood characteristics at time of diagnosis**				
African American residents (%)	14.0	15.2	0.2	75.3
Foreign-born residents (%)	27.5	13.7	4.4	54.8
Social vulnerability index (0–400)	132.9	65.8	37.2	289.0
Occupational prestige score (29–62)	37.4	3.8	31.4	48.3
Property crime rate (per 1000)	53.4	34.4	9.1	390.7
Residents who moved in the preceding 12 months (%)	26.2	10.5	6.2	55.1
Housing built before 1940 (%)	25.1	18.9	0.0	97.9
Neurological hazards index (rescaled by 100)	8.0	1.3	4.7	13.5
*N*	588			
**City and County of Denver, 2000**				
African American residents (%)	11.5	16.6		
Foreign-born residents (%)	15.8	10.7		
Social vulnerability index (0–400)	96.7	42.0		
Occupational prestige score (29–62)	41.1	4.4		
Property crime rate (per 1000)	48.3	47.0		
Residents who moved in the preceding 12 months (%)	27.8	12.3		
Housing built before 1940 (%)	25.1	25.7		
Neurological hazards index (rescaled by 100)	7.7	2.0		

**Table 3:** Average Marginal Effects from IV Probit Models Predicting the Probability of Ever Being Diagnosed with a Neurodevelopmental Disorder.

	Full Sample		
**Individual characteristics**			
Latina female	0.042		(0.076)
Latino male	0.073		(0.077)
African American female	0.028		(0.047)
**Parent/caregiver characteristics at time of first offer**			
PC age	0.000		(0.002)
PC immigrant	0.048		(0.054)
PC has a disability	0.124		(0.085)
PC has HS diploma or more	0.035		(0.045)
**Household characteristics at time of first offer**			
No. of siblings in household	−0.026		(0.021)
Household income at first offer ($)	0.013		(0.021)
Household stressor scale (0–5)	0.037		(0.022)
Mean number of moves from birth to time of first offer	0.019		(0.020)
**Neighborhood characteristics at time of diagnosis**			
Social vulnerability score (0–400)	0.406	***	(0.097)
Occupational prestige score (29–62)	0.183	*	(0.079)
African American residents (%)	−0.059		(0.054)
Foreign-born residents (%)	0.124		(0.072)
Residents who moved in the preceding 12 months (%)	−0.120	*	(0.059)
Property crime rate (per 1000 residents)	0.037		(0.086)
Housing built before 1940 (%)	0.185	**	(0.064)
Neurological hazards index (scaled to 100)	−0.427	***	(0.071)
*N*	588		
Wald χ^2^	206.88	***	

NOTE: * *p* < 0.05; ** *p* < 0.01; *** *p* < 0.001.

## Data Availability

The data for this study are under a Data Confidentiality Agreement with the Denver Housing Authority. Requests for the data analyses supporting the research in this study may be made to the first author.

## Supplementary Materials

The following are available online at https://www.mdpi.com/article/10.3390/ijerph18179041/s1, Supplementary materials: Description of the Denver Child Study Survey, Other Data Sources and Construction of Variables, Reference [68] is cited in the supplementary material.
