# Supplementary material for: Assessing the Impact of Neighborhood Conditions on Neurodevelopmental Disorders during Childhood
Source: Int J Environ Res Public Health. 2021 Aug 27;18(17):9041. doi: 10.3390/ijerph18179041 (PMC8430861; doi:10.3390/ijerph18179041)
Supplement: Supplementary file 1 [file ijerph-18-09041-s001.zip › ijerph-1324251-supplementary.pdf]

Description of the *Denver Child Study* Survey, Other Data Sources and Construction of Variables

Denver Child Study Household Survey

The *Denver Child Study* telephone survey was fielded during 2006-2008 (conducted in person for about 20% of the sample who had no landline phones). The survey collected retrospective and current information about the household, adults and children. Detailed information related to multiple domains of child outcomes was gathered for all eligible children associated with each household. Each household's residential mobility history was documented so it could be associated with specific neighborhood developmental contexts for children. Current and former DHA resident families were eligible to participate in the study if they met all of the following criteria: (1) presence of children in the home between ages 0 and 18 years when they moved into DHA; (2) family first entered DHA in 1987 or later (when DHA's current quasi-random assignment process came into operation); (3) family remained in DHA housing for at least two years; and (4) family was of Latino or African American ethnicity.

Attempts to recruit participants for the study were made by mail, phone and in person, in both English and Spanish when appropriate. Compensation for participation took the form of $50 in cash or a gift card. The overall participation rate was 56.5% (85% for those still residing in DHA), with most non-participation due to the inability to locate the household. Less than 6% refused to participate once contacted. A total of 711 interviews with the primary caregivers of eligible households were completed and whose surveys subsequently passed rigorous data verification and reliability checks. Approximately 1,800 children and youth analyzed in the study were current or past members of these 711 households who spent two or more years residing in DHA housing before reaching age 19.

Variables Measuring Characteristics of Caregivers and Households

The *Denver Child Study* survey collected information on a wide variety of parental/caregiver ("caregiver" hereafter) and household characteristics. This included time-invariant information about caregiver such as gender, year of birth, ethnicity and national origin.

The survey also measured many aspects of the caregiver and household that varied over the life of the child, such as educational attainment, economic status, disability status, marital status, fertility and employment histories, and access to health insurance. The survey also included a Household Stressors index comprised of five items that identified potential difficulties faced by the household in terms of finances, employment, insurance, utilities, and housing. For each year in a child's life, this index measures the magnitude of stressors facing the household at the corresponding residential location. Caregivers were asked whether they experienced any of the following events which residing at the specified residential location:

a. Unemployed a month or more?
b. Have a major illness or injury?
c. Have too little money to buy enough food for your family?
d. Have your electricity, gas, or phone service cut off?
e. Get evicted from your home?

Possible responses to each item (followed by their associated score) were no (0) and yes (1), indicating either the presence or absence of a certain stressor. Scores ranged from 0 to 5, with higher values indicating a greater degree of household stress. The Cronbach's alpha for this index was .50.

Variables Measuring Characteristics of Children and Youth

The *Denver Child Study* survey also asked caregivers to supply information about all of their children with whom they had lived in DHA public housing for at least one year. In this manner, detailed information was collected about the children's gender, ethnicity, birth order, residential histories, health, exposure to violence, behaviors and activities, education and (for older children), marital/fertility histories and labor market outcomes during early adolescence and young adulthood. The focal child's number of siblings was recorded during the time period under analysis, as well as their behaviors (e.g., dropping out of high school). Finally, residential history information facilitated the computation of the number of moves the household had undertaken during the childhood of the observed youth.

Variables Measuring Characteristics of Neighborhoods Experienced by Children and Youth

It is generally accepted that "neighborhood" has both objective "space" dimensions (i.e., economic, demographic, social indicators associated with geographies) and subjective "place" dimensions (i.e., the human experience of territory) [68]. The study obtained a wide array of neighborhood data about both dimensions from three sources.

The first source was the decennial U.S. Census, where census tract geographic scales from 1970, 1980, 1990 and 2000 censuses were utilized. The main source of these data was the *Neighborhood Change Data Base* (a Geolytics proprietary product) a longitudinal neighborhood file that adjusts data to account for changes in tract boundaries between decennial censuses. For estimates of non-census year data, linear interpolation or extrapolation was employed. Indicators that have been widely employed in prior research on neighborhood effects were compiled, including percentages of households moving in during the prior year, female-headed households, families below the poverty line, unemployed adults, Latino population, non-Latino African American population,[1] foreign-born population, homes that are renter-occupied, homes that were built during various periods, and mean occupational prestige based on the General Social Survey prestige score weighted by the observed proportional distribution of occupations of employees in the tract. Given high correlations among several of these variables, we four principal components analyses were conducted, one for a comparable set of variables for each of the 1970-2000 censuses.[2] For each census year, the analysis produced a single component (with an eigenvalue greater than unity) that consistently was comprised of the roughly equally weighted sum of census tract percentages of: poor, unemployed, renters, and female household heads. This became the neighborhood social vulnerability score (ranging from 0-400) used in the study.

The neighborhood social vulnerability score used in the *Denver Child Study* is not strictly comparable to similar indices of "neighborhood disadvantage" used in many prior scholarly works for two reasons. First, the index sums neighborhood percentages of unemployment, poverty, female-headed households and renters; it does not include ethnic, racial, or nativity measures, as do most others. Second, the models we employ in our analyses control for a host of other neighborhood characteristics that are often associated with "disadvantaged neighborhoods" but for which other studies have no direct measures such as occupational prestige. Thus, other studies' "neighborhood disadvantage" variables serve as ambiguous proxies for a diverse bundle of conditions and

[1] The ethnic makeup of Denver in 2000 was 52 percent non-Latino whites, 11 percent non-Latino African Americans, and 32 percent Latinos.

[2] The creation of our linked database occurred prior to the release of the 2010 Census and the five-year average *American Community Survey* data.

quantitative results using such measures should not be used as precedents for results using this social vulnerability measure.

The second source of neighborhood information was the Denver-based Piton Foundation's *Neighborhood Facts Database,* which provided small area-based, annually measured information culled from Denver administrative databases on characteristics that are not provided by the Census. These included violent crimes reported to police per 1,000 population, property crimes reported to police per 1,000 population, and confirmed cases of child abuse and neglect per 1,000 children. The Piton Foundation data are aggregated to 77 named community areas consisting of two census tracts, on average, and thus are measured at a larger spatial scale than our census-based data. Moreover, Piton series data before 2018 were available only for the City and County of Denver, which produced shrinkage in the analysis sample because some former DHA households that were interviewed had moved out of the county.

The third source for data on toxic airborne pollutants coded to census tracts was from the U.S. Environmental Protection Agency. In particular, their summary index of respiratory health risk associated with the combined concentrations of 124 toxic airborne compounds as well as their neurological risk index identifying the concentrations of lead pollutants were utilized. The respiratory and neurological risk indices are generated from tract-level estimates of 124 air toxins (listed at http://www.epa.gov/ttn/atw/nata2002/02pdfs/2002polls.pdf). These estimates are then aggregated based on a 'risk per million' index, with the number representing the likelihood that one person, out of one million equally exposed people, would develop the respiratory or neurological health issue if exposed continuously to the specific concentration over a 70-year lifetime; source and further information: http://www.epa.gov/ttn/atw/nata2002/natafaq.html#A6. In our analyses, we have rescaled the neurological risk index by a factor of 100.

Quality Controls and Creation of Analytical Databases

Information provided by respondents on their residential histories was cross-checked with residential location information available in the DHA administrative databases, U.S. Postal Service, Lexis-Nexis and Intelius address files as well as several additional online search engines. Once residential history information obtained on the survey was verified for accuracy, each address was geocoded using the U.S. Bureau of the Census' *American FactFinder* website utility. In cases where respondents could not recall specific addresses but only proximate cross-streets, these locations were verified using MapQuest and then identified the corresponding census tract using the aforementioned Census website showing tract boundaries. This procedure provided the census tract corresponding to each location in respondents' residential histories, which, in turn, permitted the matching of each location to the aforementioned battery of neighborhood indicators for census tract neighborhoods. These techniques allowed for the successfully matching of 92 percent of the residential locations identified by respondents.

These data for households and neighborhoods were then transformed into the format of a *child-year unit of observation*. For each child-year there are variables associated with: (1) fixed child characteristics, (2) fixed caregiver characteristics; (3) temporally varying child characteristics; (4) temporally varying caregiver-household characteristics; (5) temporally varying neighborhood characteristics; and (6) temporally varying outcomes.

Reference

Fitzpatrick, K.,; LaGory, M. *Unhealthy Places: The Ecology of Risk in the Urban Landscape;* Routledge: New York, NY, USA, 2000.
